# 

**Published:** 1960-03

**Authors:** 


### THE MEDICAL JOURNAL OF THE SOUTH-WEST

*Incorporating*
*The Bristol Medico-Chirurgical Journal*

NATIONAL LIBRARY
MAY 11 1960
MEDICINE

*March 1960*

